# Efficacy of intra-lesional injections of meglumine antimoniate once a week *vs*. twice a week in the treatment of cutaneous leishmaniasis caused by *L*. *tropica* in Iran: A randomized controlled clinical trial

**DOI:** 10.1371/journal.pntd.0010569

**Published:** 2022-07-08

**Authors:** Amir Javadi, Ali Khamesipour, Mohammad Ghoorchi, Mahdieh Bahrami, Alireza Khatami, Iraj Sharifi, Seyed Ebrahim Eskandari, Alireza Fekri, Mohamad Reza Aflatoonian, Alireza Firooz

**Affiliations:** 1 Community Medicine Department, Qazvin University of Medical Sciences, Qazvin, Iran; 2 Center for Research and Training in Skin Diseases and Leprosy, Tehran University of Medical Sciences, Tehran, Iran; 3 Mashhad Health Network, Mashhad University of Medical Sciences, Mashhad, Iran; 4 Bam Health Network, Kerman University of Medical Sciences, Kerman, Iran; 5 Leishmaniasis Research Center, Kerman University of Medical Sciences, Kerman, Iran; 6 Research Center of Tropical and Infectious Diseases, Kerman University of Medical Sciences, Kerman, Iran; 7 Clinical Trial Center, Tehran University of Medical Sciences, Tehran, Iran; U.S. Food and Drug Administration and Center for Biologics Evaluation and Research, UNITED STATES

## Abstract

Treatment of Cutaneous leishmaniasis (CL) is based on using antimoniate derivatives; patients’ compliance for systemic injections is low due to the pain and systemic complications. In this randomized open trial, the efficacy of intra-lesional (IL) injections of meglumine antimoniate (MA) once a week *vs*. twice a week in the treatment of Anthrpoponothic CL caused by *L*. *tropica* was studied. Eligible volunteer patients were selected according to inclusion/exclusion criteria. The included patients were randomly allocated to receive IL-MA injections once a week or twice a week. The primary outcome was set as complete healing of the lesion(s), and defined as complete re-epithelialization and absence of induration in the lesions. A total of 180 parasitologicaly proven CL patients caused by *L*. *tropica* were recruited, 90 patients were treated with weekly IL-MA and 90 patients received IL-MA twice a week. The complete cure was 87.9% vs. 89.2% in the group received weekly and twice a week IL-MA injections, respectively (P = 0.808). Patients’ compliance was acceptable and side effects were limited to a few local allergic reactions to MA. Median time to healing was significantly shorter in patients who received IL-MA twice a week (median ± SE) 37±3.8, (CI: 29.6–44.4) days compared to whom received IL-MA once a week 60±2.3, (CI: 55.6–64.5) days (P< 0.001), however the number of injections was higher in group who received IL-MA twice a week (12 vs. 9 injections). In conclusion, the rate of cure in the group of CL patients with IL-MA twice a week was not significantly different from the group who received IL-MA once a week shorten, but the duration of healing was shorter in the group who received IL-MA twice a week while the group received more injections so is recommended to use IL-MA once a week due to the fact the compliance is acceptable with limited side effects.

**Clinical Trial Registration:** IRCT20081130001475N13; https://en.irct.ir/.

## Author summary

Cutaneous leishmaniasis (CL) is an emerging uncontrolled neglected tropical disease which annually affecting millions of people in more than 80 countries. Currently available control strategy measures of CL showed not to be fully effective. Although, antimoniate derivatives are in use to treat CL for the last 80 years, but treatment of CL is a dilemma. Systemic use of antimonite derivatives is painful, with limited compliance and accompanies severe adverse events. Efficacy of intra-lesional injections (IL) of antimoniate derivatives is similar to systemic treatment, but there is no agreement how to use IL antimonite derivatives. In the present study efficacy of IL use once a week was compared with twice a week in the treatment of ACL caused by *L*. *tropica* in 180 parasitologically proven CL patients. The results showed that the duration of healing is shorter in those who received Glucantime twice a week, but overall treatment of CL using IL once a week is superior to twice a week and more practical.

## Introduction

Leishmaniasis is endemic in 98 countries and territories, currently about 10% of the world population are at risk of infection, annual incidence of the disease is 1.5–2 million cases. Cutaneous leishmaniasis (CL) is the most common clinical form of leishmaniasis and is endemic in more than 80 countries. Ninety percent of CL cases are reported from Afghanistan, Brazil, Iran, Peru, Saudi Arabia and Syria, CL is a major health problem in Middle East [1–3]

Both form of Old World CL, zoonotic CL (ZCL) caused by *L*. *major* and anthroponotic CL (ACL) caused by *L*. *tropica*, are endemic at least in 18 provinces of Iran and is a major health problem in the country. Two major ACL endemic cities are Mashhad (Khorasan Razavi province) and Bam (Kerman province). The number of ACL patients in 2003 in Khorasan province was 5,399, from those 3,673 cases were reported from city of Mashhad. In 2009, Khorasan Razavi with 5,860 CL cases was the most infected province in the country. Bam is another endemic focus for ACL with incidence of 7.6 per 1,000 during 2004–2008 [3–7].

Although CL is a self-healing lesion, the healing process takes a long time and treatment of CL especially ACL is a major challenging issue in endemic areas. Since 1929, antimoniate derivatives meglumine antimoniate (Glucantime) and sodium stibogluconate (Pentostam) are the only standard treatment for CL.

In different parts of the world, various modalities are used to treat CL with controversial results. Due to anthroponotic nature of ACL, treatment is probably the most effective control measure. Iranian national guideline for the treatment of CL recommended to treat every lesion caused by *L*. *tropica* either by systemic injections of MA 20 mg antimony (Sb5+)/kg body weight for 21 days (at most 3 ampoules per day) or weekly IL-MA injections. However, when systemic injections are used the patients’ compliance is poor which might be a reason for emerging resistance. Treatment is not safe and not suitable for patients with kidney, heart and liver health problems [5,6,8–12]. According to systematic review published articles, regardless of heterogeneity and low quality of the included studies, the overall efficacy of CL treatment with MA was more than 70%, and was concluded that the efficacy of IL injections is similar to systematic injections, the authors could not conclude which regimen is more effective [13,14].

The most efficacious IL injections of MA treatment schedule and timing is not well defined yet, so in the current study the efficacy of weekly injections of IL-MA was compared with twice a week injection of IL-MA in the treatment of ACL caused by *L*. *tropica* in two major endemic areas of ACL in Iran.

## Materials and methods

### Ethics statement

The protocol (protocol number: J/423/2252) of the study was approved by Institutional Ethical Committee of Center for Research and Training in Skin Diseases and Leprosy, Tehran University of Medical Sciences, Tehran, Iran. The study was conducted according to the Good Clinical Practice (GCP) guidelines and was register in Iranian Registry of Clinical Trials (IRCT20081130001475N13) (https://en.irct.ir/).

### Study design and population

This study was designed as an open randomized controlled clinical trial with two parallel arms. Two ACL endemic areas of Mashhad and Bam with enough ACL patients were selected and the study was completed.

### Inclusion criteria

Patients with clinically suspected CL lesion(s) were screened. Parasitological proven (smear and/or culture) CL patients caused by *L*. *tropica* were recruited into the study if they met other eligibility criteria. Polymers chain reaction (PCR) was performed on every collected sample to assure that the lesion was caused by *L*. *tropica*. Other inclusion criteria were age 8–70 years, willingness to participate in the trial, and sign an informed consent and an oral assent from the children.

### Exclusion criteria

Patients with lesion(s) duration more than 6 months, more than 4 lesions, ulcer size more than 3 cm, lesions on the face or close to a vital organ, pregnant and nursing patients, those with a history of previous systemic or IL treatment with MA or those with an acute or chronic disease that could affect the course of CL or treatment with IL-MA injections were excluded.

### Location and participants

Mashhad is an ACL endemic city and the capital of Khorasan Razavi province which is located in northeast of Iran close to the borders with Afghanistan and Turkmenistan. Mashhad is the second most populated city in Iran with more than 3 million populations and is a pilgrimage city as well. Millions of pilgrims from all over the world visit the city every year. The city of Bam is in Kerman province, southeast Iran with 100,000 populations and is endemic to ACL.

### Interventions

The patients were randomly assigned to either receive IL injections of MA (Glucantime, Sanofi-Aventis, France) once a week or twice a week. IL-MA was injected into each lesion using fine 30G needles until, the lesion was completely blanched: 0.2–1.5 ml per lesion depending on the size of lesion, each treatment continued up to 12 weeks or completes healing of the lesions whichever occurs earlier. Lesions characteristics including sizes of induration, ulcer and scar were measured and recorded weekly before IL-MA injections and during the treatment according to the method which was described earlier [15,16].

### Study outcomes

The primary outcome of the study was complete cure defined as complete re-epithelialization of the lesion with no induration. The secondary outcome measures were the time-to-complete cure of the lesion(s) and the proportion of the adverse events in each treatment arm.

### Sample size

Using EpiInfo 7 (CDC, Atlanta, GA, USA) and assuming efficacies equal to 85% and 65% for those whom receive twice a week and weekly IL-MA injections, respectively; and type I error equals to 0.05, type II error equals to 0.2, a sample size equals to 70 patients for each group was calculated. To compensate for 20% estimated lost to follow up or withdrawn, 90 patients were needed for each arm of the study.

### Randomization and randomization concealment

Random sequence generation was prepared using version 16 of SPSS (SPSS Inc. Chicago, IL, USA) software. To conceal the random sequence from the recruiter, sequentially-numbered opaque sealed envelope (SNOSE) was used, briefly upon a volunteer patient agreed to participate and signed an informed consent, she/he received a SNOSE and was referred to the health care worker who assigned the volunteer to receive IL-MA injections once a week or twice a week according to the randomization list.

### Statistical methods

Shapiro–Wilk test was used to evaluate normality of numerical variables. Continuous variables were described as mean ± standard deviation (SD), if they had Normal distribution. If they had non-parametric distribution, median and interquartile range (IQR) were provided. Categorical and ordinal variables were presented as proportions. Data summaries were demonstrated in the table.

To compare the means of parametric data between the two groups, independent t test was used. Comparison of median for variables with non-parametric distribution between the groups was done using Mann-Whitney *U*-test. To compare the proportions, Pearson chi-square test or Fisher’s exact was used. For analysis of time-to-healing between the two groups, survival analysis with Kaplan-Meier method was used and for comparing the difference, log-rank test was performed. The findings were presented as median 95% confidence intervals (CI). For all tests, we used 0.05 as the significance threshold for the P value.

## Results

A total of 476 CL suspected patients (Mashhad = 252, Bam = 224) were screened and 180 (F = 82, M = 98) CL confirmed patients with 224 lesions; 96 CL patients with 109 lesions in Bam and 84 patients with 115 lesions in Mashhad were recruited in the study. The baseline characteristics of the two groups including the number of lesions, size of induration and ulcer at the initiation of treatment were not significantly different (Table 1).

**Table 1:** Baseline characteristics of the patients included in the two arms.

		Dose per Week		
Variables		One Injection (n = 90)	Two Injection (n = 90)	P-Value
Gender	Female (%)	42 (46.7)	40 (44.4)	0.76
	Male (%)	48 (43.3)	50 (53.6)	
Mean of age (Year)		27.21 ± 1.69	27.12 ± 1.66	0.97
Mean of lesion number		1.19 ± 0.05	1.29 ± 0.07	0.19
Mean of induration size (cm)		5.22 ± 1.60	5.47 ± 1.29	0.20
Mean of ulcer size (cm)		2.30 ± 0.82	2.23 ± 0.91	0.49
Onset Mean of HX of Sore (Week)		10.21 ± 0.64	11.29 ± 0.75	0.27

**Fig 1:**
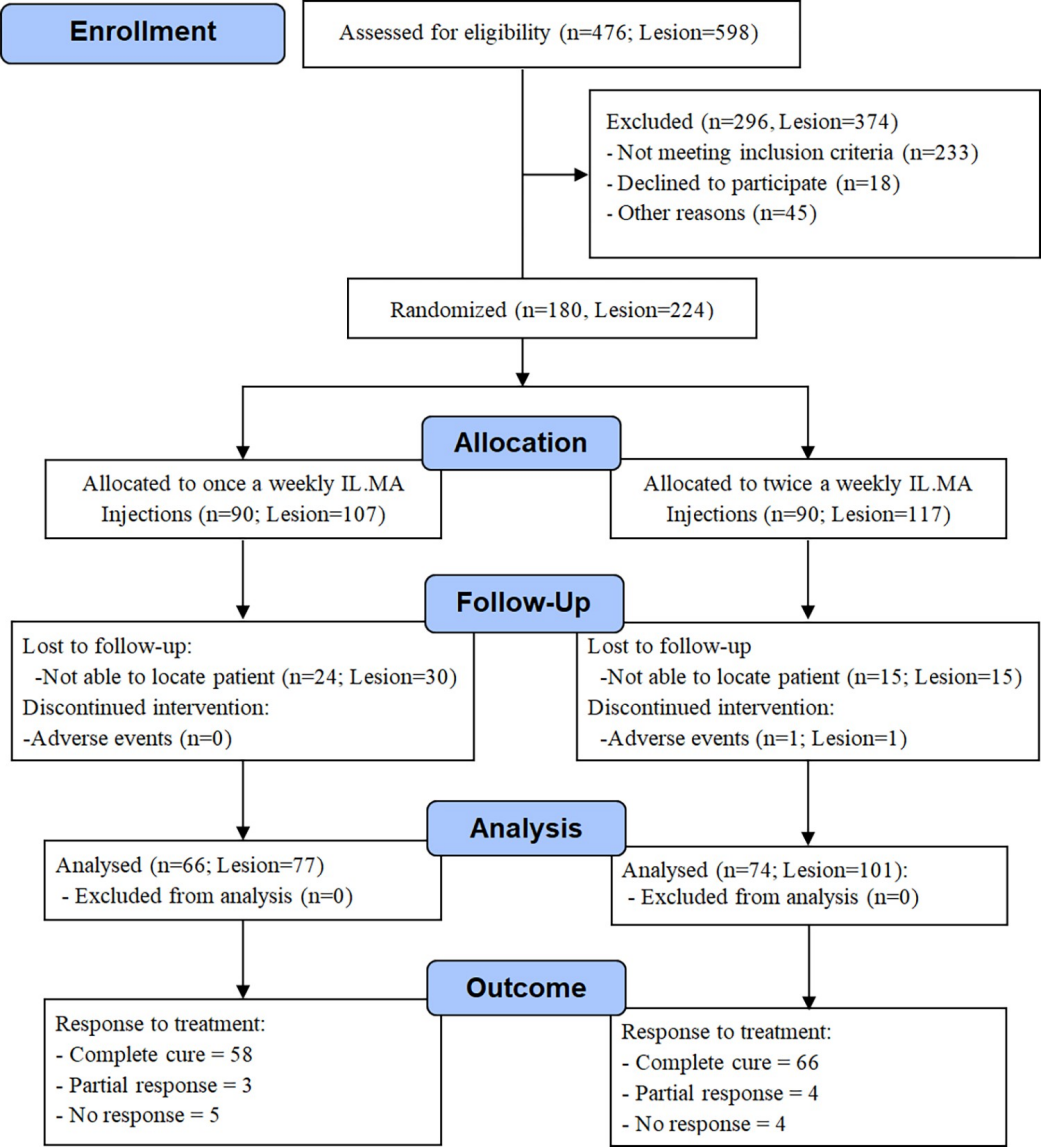
Flow diagram of patient enrollment treatment and follow up.

The recruited patients were randomly assigned to receive either once a week or twice a week IL-MA injections. Sixty-six patients with 77 lesions in the group whom received once a week IL-MA injections and 74 with 101 lesions in the group whom received IL-MA twice a week have completed the study (Fig 1).

At the end of the treatment period the proportion of complete cure was 87.9% in weekly IL-MA injections group and 89.2% in the twice a week IL-MA injections group (p = 0.808). The proportion of partial cure was in group whom received weekly IL-MA was 4.5% in comparison with 5.4% in whom that were treated twice a week IL-MA injections (p = 0.816). No response proportions were 7.6% and 5.4% in the weekly IL-MA and twice a week IL-MA injections groups, respectively (p = 0.601).

**Fig 2:**
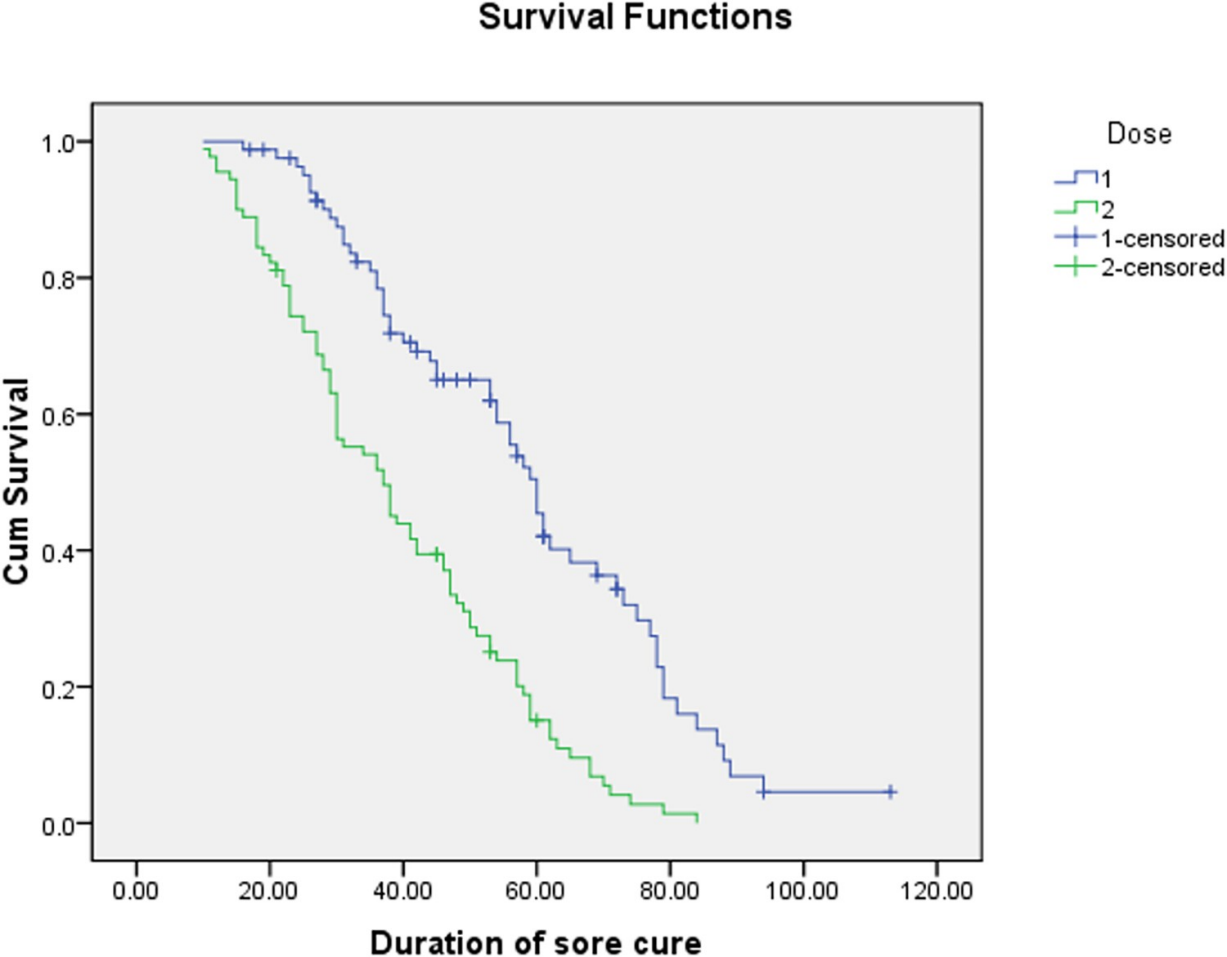
Survival function of sore cure separate by dose injection.

Duration of healing in patients whom received twice a week IL-MA injections, the median ±standard error (SE) duration was 37±3.8 (CI: 29.6–44.4) days, which was significantly shorter in comparison with the group whom received with a median of injections in group who received a median of injections weekly IL-MA injections 60±2.3 (CI: 55.6–64.5) days (p<0.001) (Fig 2). However, the number of injections was more in group who received IL-MA twice a week (12 *vs*. 9 injections). On every visit patients complain was acceptable and side effects were limited to a few local allergic reactions to MA.

## Discussion

Cutaneous leishmaniasis is a major health problem in some endemic areas, both ZCL with an incidence rate as high as 20% in some endemic areas and ACL as high as 5% in city of Bam, Kerman province are major health problem of endemic areas in Iran. Various modalities such as physical, immunological, topical and systemic agents have been used to treat CL. Pentavalent antimonials (sodium stibogluconate = Pentostam and meglumine, antimoniate = Glucantime), which are still the World Health Organization first-line treatment for CL, requires multiple injections which is painful and as such is not tolerated by most of the patients which resulted in low compliance. Moreover, antimonial derivatives are not always effective especially when causative agent is *L*. *tropica*. Most of the CL patients do not tolerate systemic injections and as such the compliance is poor, which is at least partly responsible for development of drug resistant in the endemic areas, moreover the treatment is not safe and should be used with cautious, in addition the efficacy is low [3,5,6,8,13]. Using intra-lesional injections of antimonial derivatives seems to be more practical, tolerated more by the patients and is cheaper for endemic areas.

In a study conducted in India, CL patients were treated with intra-lesional injections of SSG once a week and compared with the patients whom were treated with IL SSG injections twice a week. The latter treatment induced a significantly higher response than IL SSG once a week, although no significant difference was seen between the cure rates among the two groups thereafter, parasite identification in this study was done in a very limited number of the patients and showed that the causative agent was *L*. *tropica* [17].

In another study performed in Pakistan, a significantly higher cure rate was seen in CL patients received weekly injections of meglumine antimoniate (MA) than those received fortnightly injections of MA. The causative agent of *Leishmania* in this study was not identified [18].

According to a systematic review published in 2007, the efficacy of systemic treatment of MA in ACL patients was 10% to 65%. When complete re-epithelization of all lesions were considered as an indication of complete cure, the efficacy was even lower; 20% at 12 weeks after treatment initiation [9]. In a study performed on 710 CL patients, the efficacy rate was similar between the patients who received systemic treatment with SSG and those who received IL injection of SSG [19]

Only 1–6 IL injections of Pentostam, every 2–3 weeks was effective to treat CL lesions caused by *L. major* [20]. In a study with small sample size, CL lesions induced by *L. major* were treated by either IL injections of MA every 3 days or every 7 days and no significant difference was seen in the rate of cure between the two groups [21]. In one study, 96 CL patients with 129 lesions were treated with Pentostam either daily, every other day, or weekly, initially 3 injections was scheduled, the clinical response was more in weekly injections than daily or every other day injections, though the onset, the causative agent and the follow up was not uniform [22]. Using intra-lesional injections of antimoniate derivatives is safer than systemic treatment of either Glucantime or Pentostam, cheaper, more comfortable for the patients with higher compliance, IL treatment especially could be benefited the patients with a few lesions, elderly patients and patients with abnormal liver, heart and kidney.

## Conclusion

In this study, the rate of cure and partial cure in CL patients who received IL-MA twice a week was not significantly different from the group who received IL-MA once a week, but the duration of healing was significantly shorter in the group who received IL-MA twice a week with more injections in comparison with the group who received IL-MA once a week (37±3.8 *vs*. 60±2.3). In endemic areas of CL especially with limited infrastructure, it is recommended to use IL-MA once a week due to the fact that the injections is painful and time consuming, moreover since the first line treatment of CL is antimonial derivatives and the more antimonial injections is used the more possibility to develop allergic reaction to antimonials is increased, using IL-MA once a week is more practical with less allergic to antimonials.

## Supporting information

S1 Checklist CONSORT 2010 checklist of information to include when reporting a cluster randomized trial. (PDF)
